# Mount Vernon Hospital for Consumption

**Published:** 1903-06-06

**Authors:** 


### MOUNT VERNON HOSPITAL FOR CONSUMPTION.

Viscount Knutsford presided at the festival dinner of the Mount Vernon Hospital for Consumption and Diseases of the Chest held at the Hotel Cecil on the 20th instant. There was a large attendance, the fact that two other hospital dinners were also being held that evening notwithstanding. Among the guests were Mr. Frank Debenham, the Hon. Sydney Holland (son of the chairman), Sir Herman Weber, Mr. F. A. Bevan, Sir Spencer Maryon-Wilson, Mr. B. L. Cohen, M.P., Sir Henry Burdett, Mr. Henry Clarke, J.P., and Mr. C. D. Rudd.

In proposing the toast of "Prosperity to the Hospital," Lord Knutsford recalled the fact that in 1886 he had had the honour of presiding at one of their dinners. He found himself that evening in the happy position of a chairman of a company who was able to assure a meeting of its shareholders of the continued and satisfactory progress of their company, and to promise them a large dividend. Their dividend, however, would not be in cash but in what he was sure would give them even greater satisfaction, the prosperity and success of the hospital. As to their progress, he had only to point to the contrast between the state of things in 1886 and now. Then the number of beds did not exceed 35. In 1895 the central block was opened and the number increased to 75, and when their east wing was completed they would have accommodation for 150 patients. But that was not all. Owing to the munificent gift of a gentleman the hospital had been able to acquire a property of 104 acres near Pinner, for a country branch, which would give them room for 100 more. And in addition to that, the accommodation for their out-patients in London being very inefficient, cramped, and uncomfortable, they had spent some £4,700 on their out-patient department in Fitzroy Square, in the midst of the homes of the poor people who used it, and had thereby achieved a vast improvement. Then another great improvement had been accomplished. The arrangements for their nursing staff had to be carefully considered, for they could not hope to secure good nurses unless they provided them with good accommodation and with rooms where, after their work, they could have that quiet that was so essential to their well-being. In 1886 their accommodation for their nursing staff was elementary, or he might even say infantile. Now the rooms had been improved; they had a pleasant dining-room and sitting-room, and a house had been taken outside, which accommodated 17 nurses. But when their new wing was completed, if they could find the money, they hoped to be able to accommodate all their staff comfortably within their own grounds. He thought he had said sufficient to justify his announcement of a dividend of successful progress. Their success was due to the great help given them by the public outside, to the unceasing attention of their professional staff, to their matron and the excellent body of nurses under her, and to the wise administration of the chairman and committee of the hospital. This success justified them in their appeal for continued help to maintain the work at its present high level, and they also wanted an additional £9,000 for the new wing because they could not accommodate more than 55 per cent. of those who applied for admission. There were at the present time 260 appropriate applicants waiting for admission. Then they wanted £3,000 for the nurses' home. These were their absolutely urgent needs, and beyond that they wanted an out-patient and a store department at Hampstead, and when the new branch at Pinner was opened they would want money for its maintenance, though it had been partly endowed by the munificence of the same gentleman who had provided the money for the building. He hoped he had impressed them with the fact that a good deal of help was required. Of course the claims on the charitably disposed were unceasing. Every post brought applications for assistance—from clergymen, and from every kind of charitable society, and it was not surprising, therefore, that a man was a little puzzled as to the direction his gifts should take. He should like to give everyone the advice of Lord Derby, not to give to individuals unless they were personally known, because the relief of individual distress was apt to do more to pauperise than to help. But on the other hand, the man who gave to a public and well-managed institution might be sure that his money would be well expended, and so might refuse all other applicants. There was no work to compare with that of the hospitals—open to persons of all nationalities, and of all creeds and politics, or of none. Suffering was the only passport of admission to their wards, where the patient received the best of advice, treatment, nursing, and food—advantages which, outside, the rich even could not always secure, but which for the poor were impossible. They could not pay for doctors, and thousands and hundreds of thousands were thus deprived of a chance of recovery. Yet the maintenance and welfare of their families depended on their recovery. It was marvellous how patiently they struggled against their manifold disadvantages, their patience being only equalled by their gratitude when relieved by the hospital. Well, then, to his friend who was puzzled to know where to bestow his gifts the question presented itself, which hospital? There were so many whose claims were presented to them, and he did not wish to detract from others, but he would commend to their careful consideration those hospitals which dealt with special diseases like consumption—that terrible scourge which hung like a dark thunder cloud over the prosperity of the family—and amongst these specially he would plead for the Mount Vernon Hospital, which, from its situation and surroundings, had the means of securing that open-air treatment which was being so favourably recommended to their attention. He was the son of a doctor and the grandson of a doctor, but he was only a layman, and could not himself speak with authority on the subject, but he would recommend everyone to read an article in The Hospital of April 25th, by Dr. Kelynack, who was one of their staff. From that they would learn that if the disease were grasped at a sufficiently early stage a cure might be hoped for, and in later stages, if not an absolute cure, great relief and prolongation of life. Man had been called a fighting animal. They ought to justify that appellation, not by fighting foreign nations, or for the expansion of the Empire, but by fighting against disease and suffering, to restore fathers and mothers to their families and to save children from growing up maimed and incapacitated for the battle of life. And as the State would not help them they must look to the public to see that they did not lack ammunition. He was quite sure that when the great value of the work they were doing was known the public would see that their ammunition was not exhausted, but would enable them not only to keep up but to extend the fight.

Mr. Benjamin A. Lyon, the Chairman of the Committee of Management, acknowledged the toast, and in doing so quoted instances of the way they had been able to render most valuable assistance to those who had sought their help. He pointed out that the grants they were getting from the King's Fund, the Sunday Fund, and the Saturday Fund were guarantees that in giving money to that hospital they were giving it to people who would use it well. All connected with the hospital were of one mind and were banded together for the glory of God and the good of men.

The Secretary, Mr. W. J. Morton, announced subscriptions amounting to £2,400; and after Mr. Cohen, M.P., had proposed the health of the medical staff, to which Dr. J. Edward Squire responded, and Mr. F. A. Bevan had briefly but cordially proposed the toast of "The Chairman," which was accorded musical honours, the proceedings terminated.

